# Postcardiac injury syndrome, peripheral hematoma of ascending aorta, and cerebral infarction after PCI: a case report

**DOI:** 10.1186/s12872-020-01608-9

**Published:** 2020-07-03

**Authors:** Yuan Wang, Wenkui Li, Chi Yuan, Hegui Wang

**Affiliations:** Department of Cardiology, Yijishan Hospital of Wannan Medical College, Wuhu, China

**Keywords:** Postcardiac injury syndrome, Peripheral hematoma, Cerebral infarction, Percutaneous coronary intervention, Prednisone

## Abstract

**Background:**

Postcardiac injury syndrome (PCIS) is an inflammatory response syndrome characterized by pericardial effusion with or without pleural effusion; however, serious PCIS with peripheral hematoma of the ascending aorta and acute cerebral infarction after percutaneous coronary intervention (PCI) have not been reported.

**Case presentation:**

This article reports a very rare case of a 40-year-old patient who developed acute pericardial and pleural effusions (both bloody), acute respiratory distress, peripheral hematoma of the ascending aorta, and acute cerebral infarction after PCI. The patient’s ECG showed bow-back downward ST elevation in leads I, II, III, and V4–V6. A blood test showed significant increases in eukaryotic-cell count, erythrocyte sedimentation rate (ESR), and C-reactive protein (CRP). Echocardiography and pulmonary artery computed tomography angiography (CTA) showed a large amount of pericardial effusion and pleural effusion. CTA of the thoracic and abdominal aorta showed a peripheral hematoma of the ascending aorta. A cranial computed tomography (CT) showed cerebral infarction anterior to the anterior horn of the right ventricle. After tracheal intubation, ventilator breathing support, pericardial and pleural drainage, and adrenocortical steroid (prednisone) treatment, he gradually recovered and was discharged 20 days later.

**Conclusion:**

We report the management of a case of serious PCIS with peripheral hematoma of the ascending aorta and acute cerebral infarction after PCI. Early diagnosis, early differential diagnosis, and early use of steroid therapy are the key in treating PCIS.

## Background

Postcardiac injury syndrome (PCIS) is an inflammatory response syndrome characterized by pericardial effusion with or without pleural effusion that is caused by cardiac injuries, usually myocardial infarction (MI) or cardiac surgeries [1, 2]. In recent years, with rapid advancement in the number, difficulty, and techniques of interventional cardiovascular procedures, such as cardiac pacemaker implantation [3], atrial-fibrillation radiofrequency ablation [4], transcatheter aortic-valve implantation (TAVI) [5], and percutaneous coronary intervention (PCI) treatment [6], postoperative PCIS has been reported. Although previous incidences of PCIS have been discussed, there have not yet been reports of PCIS accompanied by peripheral hematoma of the ascending aorta and acute cerebral infarction after PCI.

## Case presentation

**Fig. 1:**
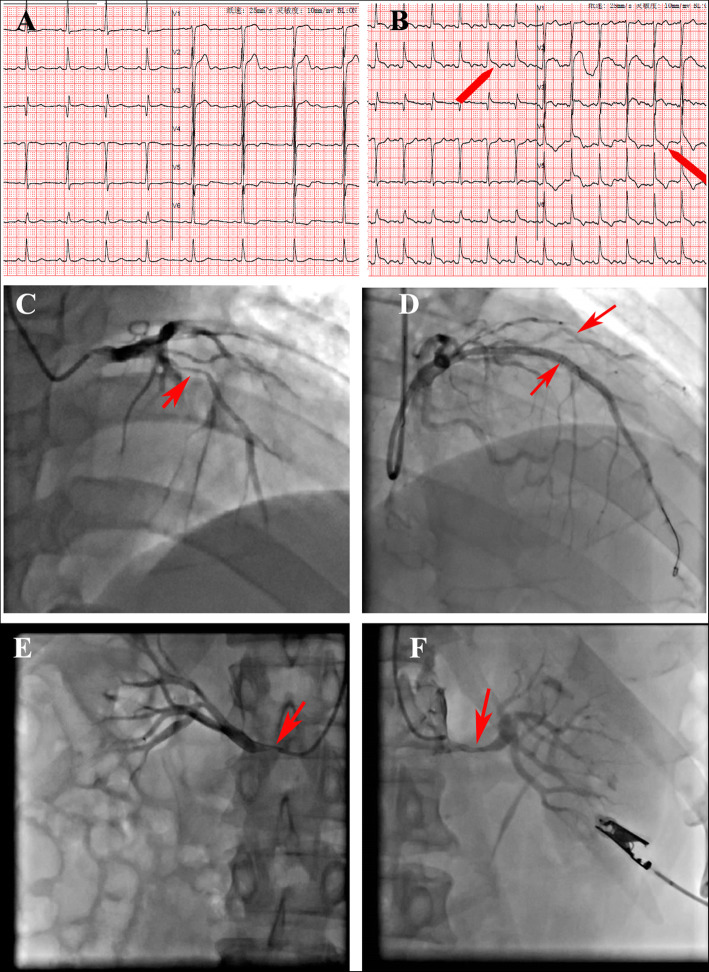
Dynamic changes in ECG and the results of coronary angiography before and after PCI. **a** Before PCI. Lead III is QR type, and leads V4–V6 show ST horizontal depression and inverted T wave. **b** Third day after PCI. Leads I, II, III, and V4–V6 show bow-back downward ST elevation (red arrow). **c** Severe stenosis in the proximal and middle segments of the LAD before PCI (red arrow). **d** The results of two stents implanted in the LAD. **e** Stenosis in the right renal artery (red arrow). **f** Stenosis in the left renal artery (red arrow)

A 40-year-old male patient was admitted to the hospital because of poorly controlled hypertension for more than a decade, recently culminating in a month of chest tightness. Physical examination showed a temperature of 36.4 °C, pulse of 71 beats/min (bpm), respiration rate of 18 breaths/min, and blood pressure (BP) of 197/98 mmHg (1 mmHg = 0.133 kPa). Auscultation revealed clear breath sounds in both lungs, without dry or wet rales. The heart expanded downwards to the left, the heart rhythm was regular, and the heart rate (HR) was 70 bpm without pathological murmurs. ECG showed abnormal inferior-wall Q wave and ST-T change (Fig. 1a). Coronary computed tomography angiography (CTA) revealed that the left-anterior descending (LAD) artery and the right coronary artery (RCA) were 90–95% narrowed. The patient was given clopidogrel (75 mg, qd), aspirin (100 mg, qd), atorvastatin (20 mg, qn), and antihypertensive drugs (amlodipine and carvedilol) before percutaneous coronary intervention (PCI).

Coronary, renal angiographies, and PCI were performed on the radial artery path. Heparin (120 U/kg) was used during PCI. The LAD artery was 90% narrowed in the proximal and middle segments. The D1 was 95% narrowed in the proximal and middle segments. The right renal artery (RRA) was 80% narrowed at the opening (Fig. 1e), and the left renal artery (LRA) was 70% narrowed in the middle segment (Fig. 1f). A 6F EBU 3.5 guide catheter and SION wires were used. Two drug-eluting stents, Firebird 3.0 × 18 mm and 3.0 × 23 mm, were implanted in the proximal and middle segments of the LAD artery (Fig. 1d). The stent release pressure was 12–14 atm. Percutaneous transluminal coronary angioplasty (PTCA) with a Sprinter 2.0 × 15 mm compliant balloon was performed in the D1 proximal end (Fig. 1d). Angiographic results were satisfactory (Fig. 1c, d).

Three hours after PCI, the patient manifested vomiting, heavy sweating, and wet and cold limbs with irritability. An emergency bedside cardiac ultrasound indicated a small to medium amount of effusion in the pericardial cavity. Pericardiocentesis was performed, and 20 mL of bloody fluid was drawn. An emergency coronary angiography (CAG) re-examination, performed to confirm the cause of pericardial effusion, showed no coronary perforation or contrast agent leakage or retention; the amount of pericardial effusion was small, which ruled out the possibility of coronary rupture or perforation (Supplementary Fig. 1).

**Fig. 2:**
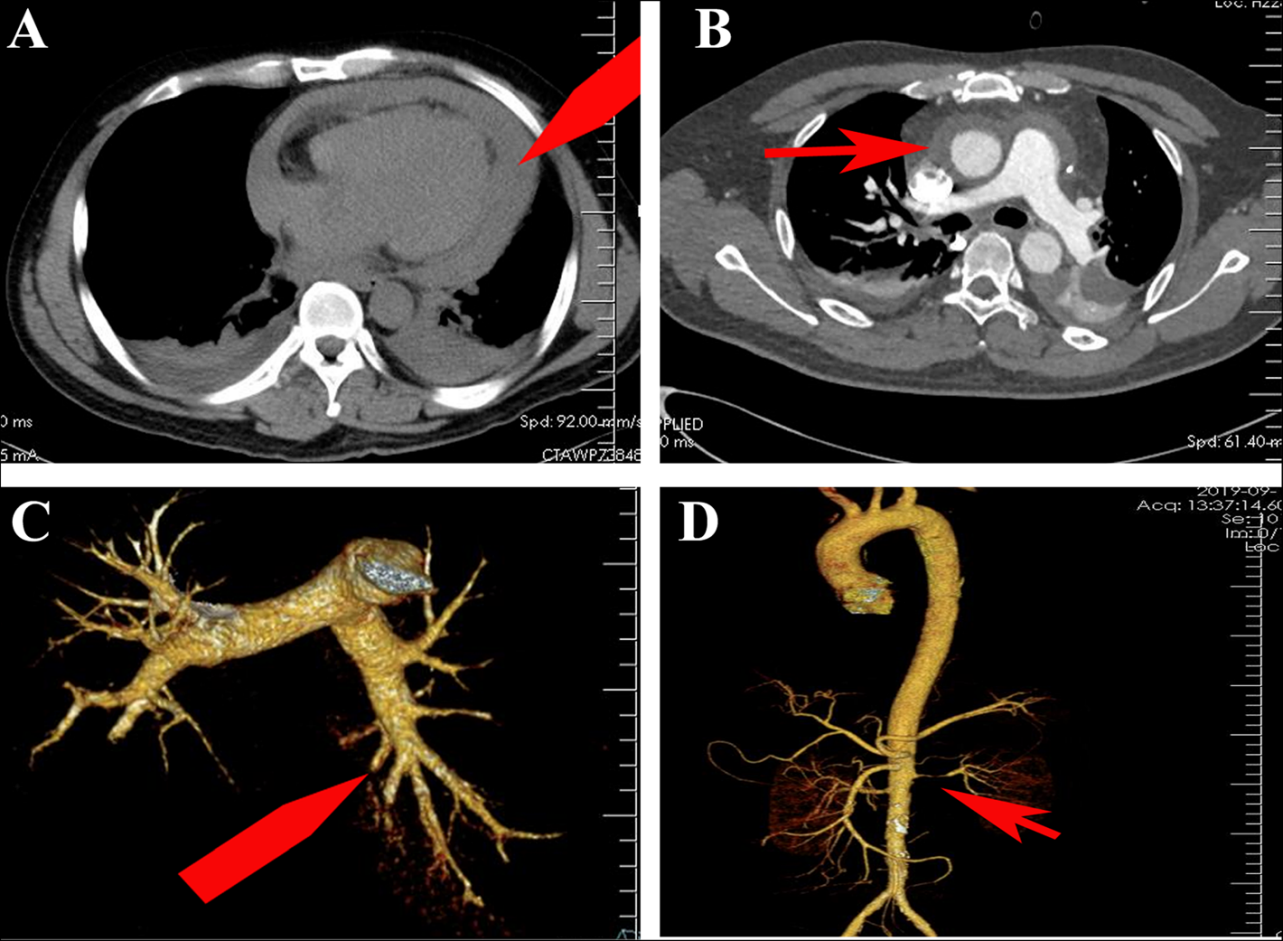
CTA of pulmonary artery, thoracic aorta, and abdominal aorta. **a** Moderate amount of pericardial effusion (red arrow) and small amount of pleural effusion. **b** Peripheral hematoma of the ascending aorta (red arrow). **c** No niche shadow, filling defect, or obvious thrombosis in the bilateral pulmonary arteries (red arrow). **d** No dissection is detected in the thoracic or abdominal aorta CTA, and stenosis of the LRA is revealed (red arrow)

On the third day after PCI, the patient was conscious with respiratory distress and hypoxemia. SaO_2_ was about 85%. Pulsus paradoxus could be palpated. ECG showed bow-back downward ST elevation in leads I, II, III, and V4–V6 (Fig. 1b). Pulmonary artery CTA showed a large amount of pericardial effusion and a small amount of pleural effusion, insufficient dilation of both lower lungs, and no significant thrombosis in the bilateral pulmonary arteries (Fig. 2). Emergency pericardiocentesis and catheter drainage were performed, and 190 mL of bloody liquid was drained. Administration of aspirin (100 mg, qd) was stopped, and the patient was only given clopidogrel (75 mg, qd).

On the fifth day after PCI, the patient’s chest tightness and pain could not be alleviated. SaO_2_ was about 85%, PCO_2_ was 28.3 mmHg, and PO_2_ was 64.3 mmHg. Biochemical test results showed an erythrocyte sedimentation rate (ESR) of 67.9 mm/h and C-reactive protein (CRP) was 231.37 mg/L (Table 1). Ultrasound results showed a small amount of effusion in the pericardial cavity and a large amount in the left thoracic cavity. We performed a left thoracic cavity puncture, and drained 500 mL of bloody fluid. The patient’s condition deteriorated, and bloody pericardial and pleural effusions occurred simultaneously. To rule out the possibility of aortic dissection, we performed an emergency CTA examination of the thoracic and abdominal aorta under tracheal intubation and mechanical-assisted ventilation. The results showed a peripheral hematoma of the ascending aorta, without dissection in the thoracic and abdominal arteries, and pleural effusion (Fig. 2). As the patient had PCIS, prednisone 20 mg/d was administered for anti-inflammatory treatment.

**Table 1:** Laboratory test data

Indicators	Pre-surgery	3 h after surgery	1st day after surgery	3rd day after surgery	5th day after surgery	7th day after surgery	20th day after surgery
WBCs (10^9/L)	9.9	17.9		15.6	15.6	13.1	12.5
NEUT%	71	65.1		77	89.5	75.9	70.1
Hb (g/L)	149	156		121	96	103	136
Cr (μmol/L)	83.2	114.3		91.7			80.2
CRP (ng/L)			15.3		231.3		3.18
ESR (mm/h)					69.7		18.8
cTnI (ng/mL)	0.03	0.01	0.04		0.14	0.09	
ALT (μ/L)	23				139	175	145
AST (μ/L)	14				63	120	49
DD (μg/mL)	0.19			1.49	1.8	3.77	

Notes: *WBCs* white blood cells, *NEUT%* neutrophil percentage, *Hb* hemoglobin, *Cr* serum creatinine, *CRP* C-reactive protein, *ESR* erythrocyte sedimentation rate, *cTnI* troponin I, *ALT* alanine aminotransferase, *AST* aspartate aminotransferase, *DD* D-dimer

**Fig. 3:**
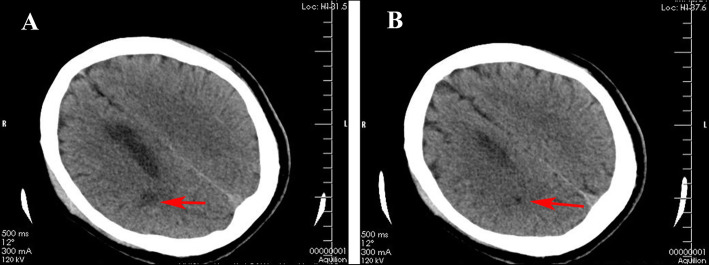
Cranial CT shows cerebral infarction anterior to the anterior horn of the right ventricle (red arrow)

On the seventh day after PCI, the patient’s complaint was decreased mobility of the left lower limb. Physical examination showed left-lower-extremity muscle power 0–1. A cranial CT showed cerebral infarction anterior to the anterior horn of the right ventricle (Fig. 3). Aspirin (100 mg, qd) was added, and dual antiplatelet therapy was used. Prednisone (20 mg/d) anti-inflammatory treatment was continued. The muscle power of the left lower extremity was restored to normal at the twentieth day after PCI. A cardiac ultrasound recheck showed no effusion in the chest and a small amount in the pericardium (supplementary Fig. 2). The prednisone was gradually reduced to 15 mg/d. After tracheal intubation, ventilator breathing support, pericardial and pleural drainage, and adrenocortical steroid (prednisone) treatment, the patient gradually recovered and was discharged 20 days later.

## Discussion and conclusions

PCIS commonly occurs after MI and open-heart surgeries [2]. Its pathogenesis is still not clear, but it is presumed to be immune-mediated in predisposed individuals that develop autoreactive reactions following the initial traumatic event. A diagnosis of PCIS depends on the syndrome’s characteristic clinical manifestations. The main features of PCIS are pericardial and/or myocardial damage, pleurisy, fever, leukocytosis, elevated ESR, serum CRP level, and high steroid reactivity [1]. Differential diagnosis should exclude acute pulmonary infarction, aortic dissection, acute MI, pneumonia, congestive heart failure, and possible malignant tumors. The clinical manifestations in this case were mostly consistent with the diagnostic criteria of PCIS, and the patient’s condition was very serious. In recent years, other instances of PCIS after PCI treatment have been reported [7].

This patient first manifested with pericarditis and was drained of a large amount of bloody pericardial effusion (1500 mL). The blood in the pericardial cavity exacerbated the development and progression of PCIS. The patient demonstrated low fever, increased eukaryotic cell count, and a significant increase in ESR and CRP. After pericardial drainage, chest drainage, and steroid drug treatment, pericardial and pleural effusions were quickly controlled, and ESR and CRP declined rapidly. In this patient, we observed a peripheral hematoma of the ascending aorta via aortic CTA, which might have been caused by a severe immune response induced by cardiac damage. The link between PCIS and ascending aortic hematoma is unclear. One - week post-PCI, the patient showed a decrease in left-lower-extremity muscle power. Cranial CT revealed cerebral infarction anterior to the anterior horn of the right ventricle. Aspirin (100 mg, qd) was immediately added and dual antiplatelet therapy was utilized. A small dose of prednisone was continuously administered. The muscle power of the left lower limb quickly recovered to normal levels, but the mechanism of cerebral infarction remained unclear. The cerebral infarction might have been an embolism, but the patient’s ECG showed sinus arrhythmia and we did not find any embolus in the left atrium by multiple echocardiography. In seeking to determine whether the cerebral infarction was an extension of the PCIS process, we searched existing literature and found no related reports. The cerebral infarction might also be related to PCIS, because the patient quickly recovered through drug therapy and the prognosis of the cerebral infarction was good.

In conclusion, to our knowledge, this is the first reported case of serious PCIS, peripheral hematoma of the ascending aorta, and acute cerebral infarction after PCI. Early diagnosis, early differential diagnosis, and early use of steroid hormone therapy are key in treating PCIS.

## Supplementary information

**Additional file 1: Supplementary Figure 1.** An emergency coronary-angiography (CAG) re-examination at 3 hours after PCI.

**Additional file 2: Supplementary Figure 2.** Cardiac echocardiography. **A** Moderate amount of pericardial effusion on third day after PCI (red arrow). **B** Tiny amount of pericardial effusion on 19th day after PCI (red arrow).

## Data Availability

The data analyzed in the case report are not publicly available due to the privacy policy of the hospital, but are available from the corresponding author upon reasonable request.

## Abbreviations

PCIS: Postcardiac injury syndrome
ECG: Electrocardiogram
CTAs: Computed tomography angiograms
CT: Computed tomography
MI: Myocardial infarction
PCI: Percutaneous coronary intervention
TAVI: Transcatheter aortic valve implantation
LAD: Left-anterior descending
RCA: Right coronary artery
CAG: Coronary-angiography
ESR: Erythrocyte sedimentation rate
CRP: C-reactive protein
