# Supplementary figures and images for: Postcardiac injury syndrome, peripheral hematoma of ascending aorta, and cerebral infarction after PCI: a case report
Source: BMC Cardiovasc Disord. 2020 Jul 3;20:317. doi: 10.1186/s12872-020-01608-9 (PMC7333298; doi:10.1186/s12872-020-01608-9)

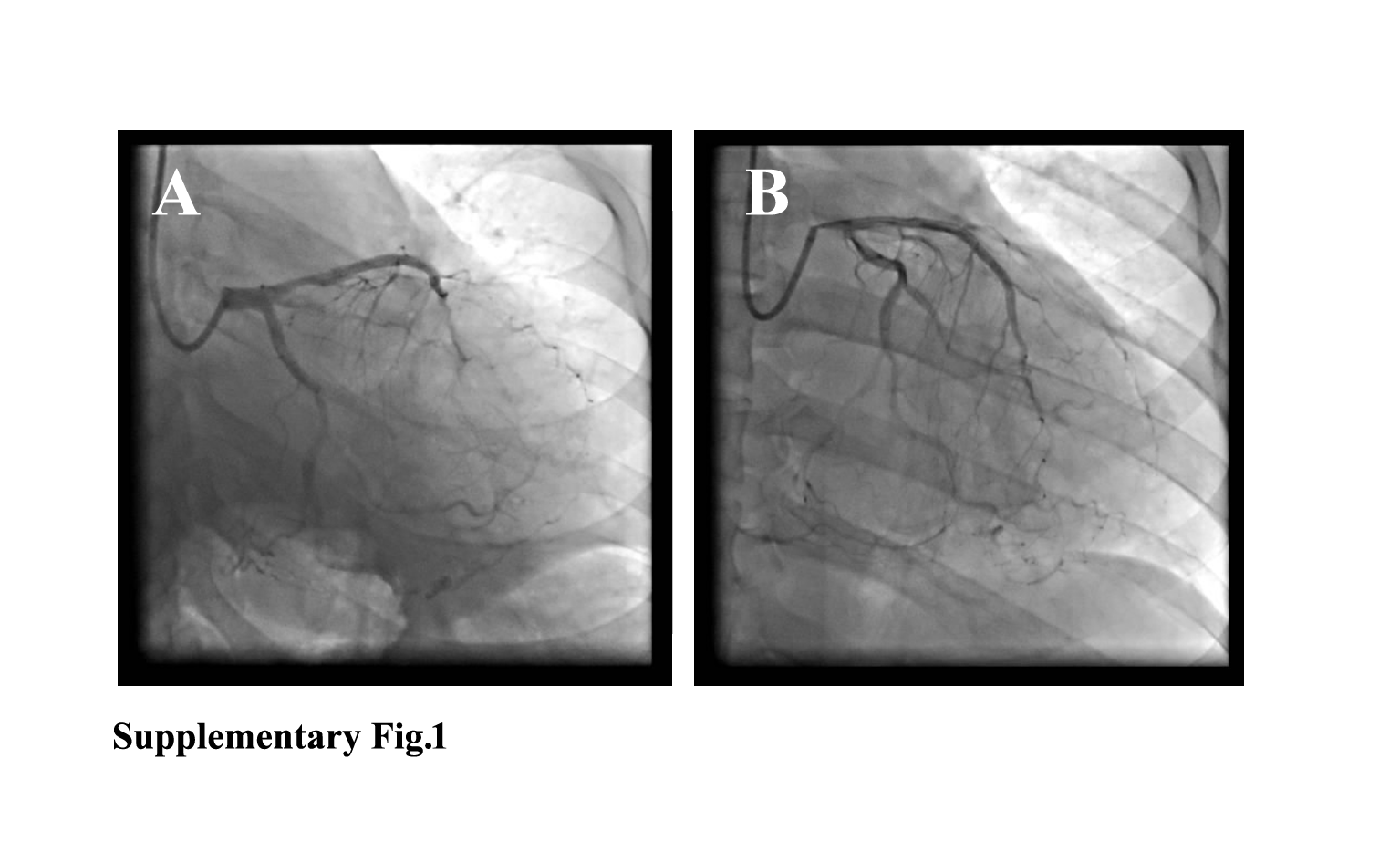

Supplement: Supplementary file 1 — Additional file 1: Supplementary Figure 1. An emergency coronary-angiography (CAG) re-examination at 3 hours after PCI. [file 12872_2020_1608_MOESM1_ESM.tif]

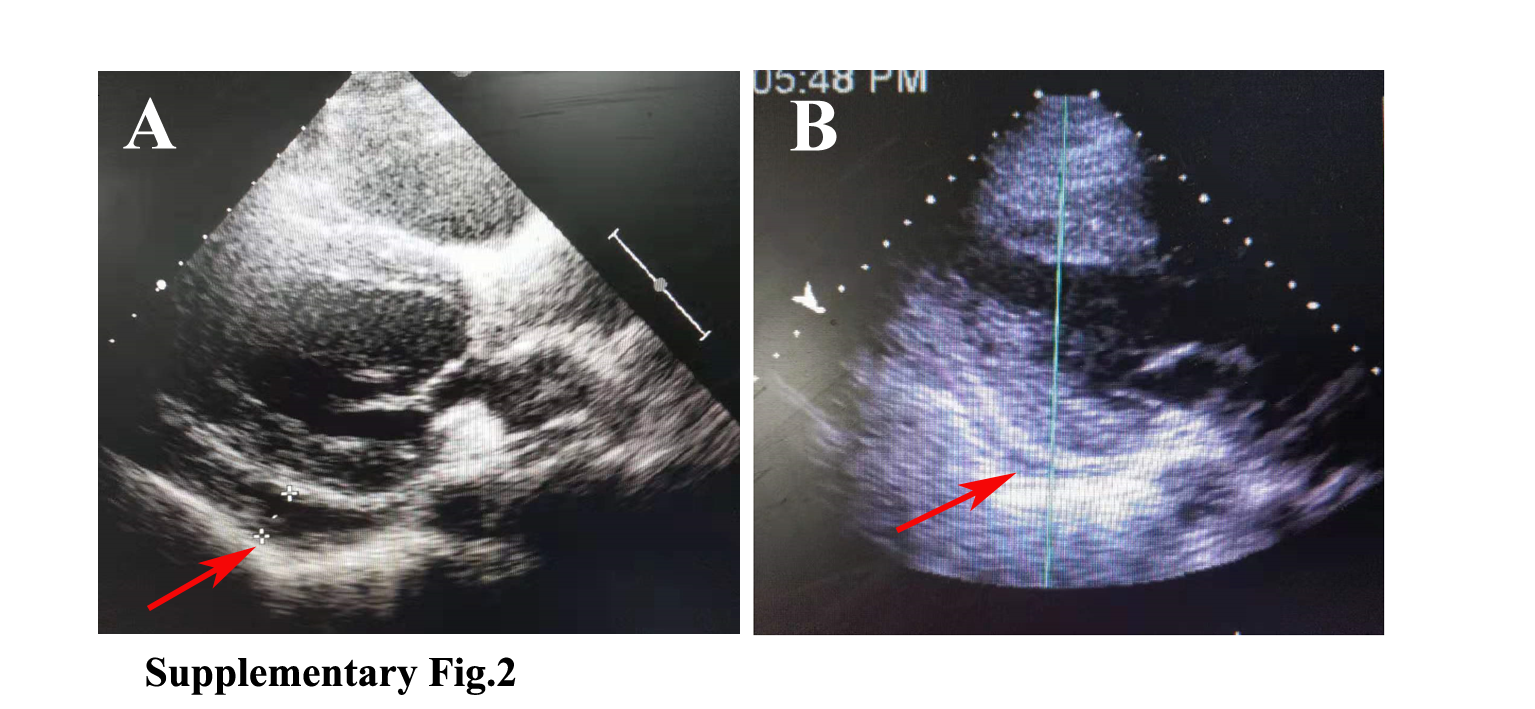

Supplement: Supplementary file 2 — Additional file 2: Supplementary Figure 2. Cardiac echocardiography. A Moderate amount of pericardial effusion on third day after PCI (red arrow). B Tiny amount of pericardial effusion on 19th day after PCI (red arrow). [file 12872_2020_1608_MOESM2_ESM.tif]
